# CancerHSP: anticancer herbs database of systems pharmacology

**DOI:** 10.1038/srep11481

**Published:** 2015-06-15

**Authors:** Weiyang Tao, Bohui Li, Shuo Gao, Yaofei Bai, Piar Ali Shar, Wenjuan Zhang, Zihu Guo, Ke Sun, Yingxue Fu, Chao Huang, Chunli Zheng, Jiexin Mu, Tianli Pei, Yuan Wang, Yan Li, Yonghua Wang

**Affiliations:** 1 Center of Bioinformatics, College of Life Science, Northwest A&F University, Yangling, Shaanxi 712100, China; 2 Key Laboratory of Xinjiang Endemic Phytomedicine Resources, Pharmacy School, Shihezi University, Ministry of Education, Shihezi, Xinjiang 832002, China; 3 Laboratory of Pharmaceutical Resource Discovery, Dalian Institute of Chemical Physics, Chinese Academy of Sciences, Dalian, Liaoning 116023, China

## Abstract

The numerous natural products and their bioactivity potentially afford an extraordinary resource for new drug discovery and have been employed in cancer treatment. However, the underlying pharmacological mechanisms of most natural anticancer compounds remain elusive, which has become one of the major obstacles in developing novel effective anticancer agents. Here, to address these unmet needs, we developed an anticancer herbs database of systems pharmacology (CancerHSP), which records anticancer herbs related information through manual curation. Currently, CancerHSP contains 2439 anticancer herbal medicines with 3575 anticancer ingredients. For each ingredient, the molecular structure and nine key ADME parameters are provided. Moreover, we also provide the anticancer activities of these compounds based on 492 different cancer cell lines. Further, the protein targets of the compounds are predicted by state-of-art methods or collected from literatures. CancerHSP will help reveal the molecular mechanisms of natural anticancer products and accelerate anticancer drug development, especially facilitate future investigations on drug repositioning and drug discovery. CancerHSP is freely available on the web at http://lsp.nwsuaf.edu.cn/CancerHSP.php.

Cancer has been declared to be one of the major global public health problems, and there is a disturbing increase in both incidence and mortality rates in recent year^1^. As a source for remedies, natural products have been recognized since ancient times and still constitute a substantial percentage of today’s new drugs and will promote drug discovery going forward^2^,^3^,^4^,^5^. To date, increasing natural products with anticancer effects were identified and isolated from herbs in last decades^6^. However, pharmacokinetics (the absorption, distribution, metabolism, and excretion (ADME) properties of a drug) and targets of these products were seldom fully evaluated owing to the time consuming and costing of the traditional methods, which have become one of major obstacles in developing novel effective anticancer agents^7^,^8^. As a holistic approach, computer-based systems approach integrates large scale experimental studies and computational analyses to provide a mechanistic understanding of drug action across multiple scales of complexity ranging from molecular and cellular levels to tissue and organism levels, leading to a new opportunity to overcome these obstacles^9^,^10^. Thus developing an anticancer natural products repository with systems methods will accelerate the development of modern medicine for cancer treatment.

Although tremendous efforts have been made in the past for providing databases containing cancer related information, to our knowledge, no such dedicated comprehensive repository of anticancer herbs and anticancer herb-originating natural products has been developed currently as yet. Some databases like CancerDR^11^ and CancerPPD^12^ have been made in the past for providing comprehensive data involved in anticancer ingredients. However, the CancerDR mainly focuses on FDA (US Food and Drug Administration) approved and experimental drugs, and CancerPPD is a database of anticancer peptides and proteins. Considering the bleak situation of cancer and absence of systematic database for anticancer herbal products, for the first time, we have developed a comprehensive repository named anticancer herbs database of systems pharmacology (CancerHSP).

CancerHSP records anticancer herbal medicines with anticancer compounds. Further more, holistic evaluation were performed for each compound. At molecular level, protein targets of the ingredients are obtained through computational predictions and literatures. At cellular level, anticancer activities of these compounds based on cancer cell lines were obtained from literatures. After mapping cell lines to primary organ or tissue sites, the therapeutic effects of each compound were emerging at the organ or tissue level. For whole body level, pharmacokinetic ADME parameters are provided by a series of computational methods. CancerHSP will not only provide more effective new therapies, but also accelerate the process of drug resistance study, drug repositioning and even anticancer drug discovery.

## Results

### Data

**Figure 1:**
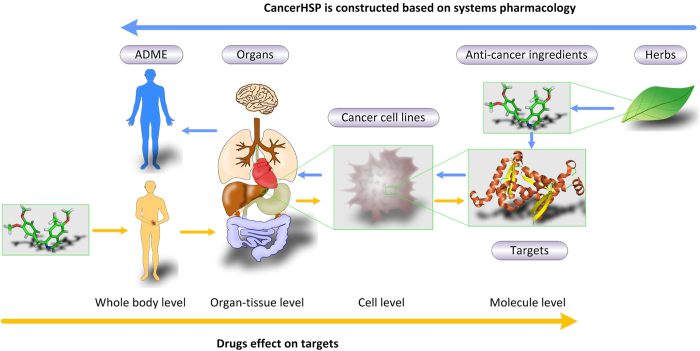
Multi-scale analyses in CancerHSP. Schematic illustration of different scales of organization involved in human pathophysiology (yellow arrows) and systems pharmacology approach (blue arrows).

As a comprehensive anticancer herbs database of systems pharmacology, CancerHSP were consisted of six major parts of data: (1) anticancer herbs, (2) anticancer ingredients for each herb, (3) targets with action mode for ingredients, (4) anticancer activities for ingredients based on cancer cell lines, (5) primary sites (organ or tissue) of cell lines, and (6) pharmacokinetic properties (ADME) of ingredients (Fig. 1). All anticancer herbs, anticancer ingredients for herbs and anticancer activities for all ingredients were compiled from research articles and book chapters.

**Figure 2:**
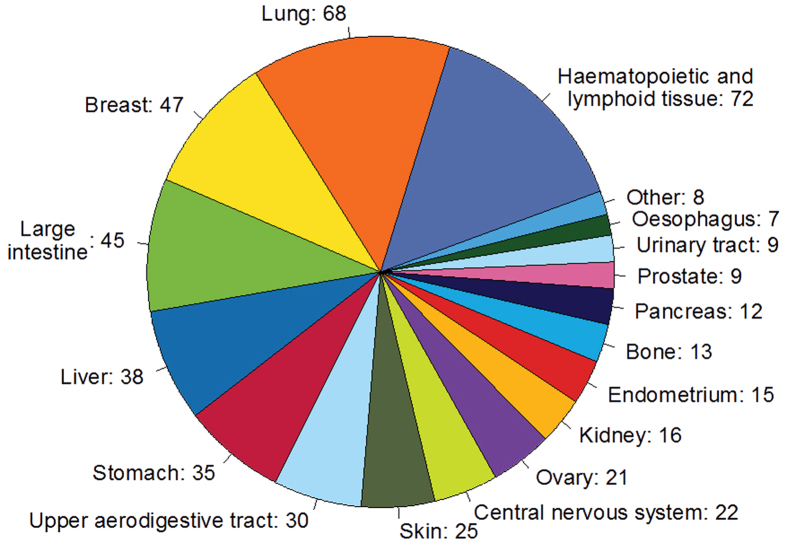
Distribution of cell lines in CancerHSP at organ or tissue level.

The current release of CancerHSP has recorded 2439 anticancer herbal medicines and 3575 anticancer ingredients coupled with anticancer activities based on 492 different cancer cell lines. These cell lines are distributed in 21 tissues and organs (Fig. 2). Further, 13 protein targets on average and nine key ADME parameters for each ingredient are predicted by state-of-art methods (see methods).

### Database description and utility

#### Search

**Figure 3:**
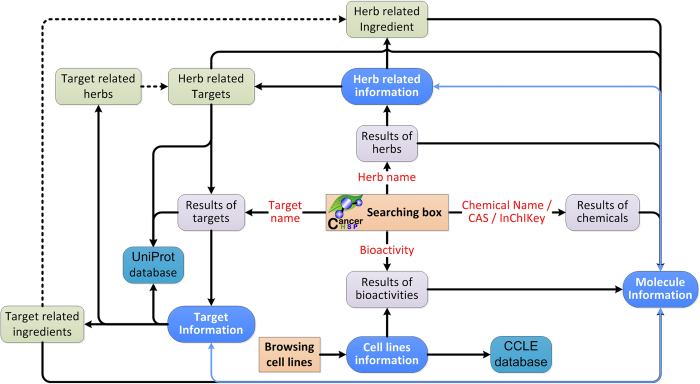
Architecture of CancerHSP interface. Boxes with the same color denote the same kind of pages.

In our web server, we have provided a user-friendly search tool for exploring CancerHSP. Users can search the information by herbal name, chemical name, InChIKey, CAS number, target name, and bioactivity in the search box at the CancerHSP homepage (Fig. 3). The results in search options come in the form of a table, which displays details in initial option as selected. For example, when users select “Chemical name” option in the search box and use “Taxol” as a keyword for searching, the browser will display the page of chemical entries which contains the highlighted “taxol” substring in the chemical names. Particularly, CancerHSP provides a very useful gadget which is a feasible way to filter and sort data, thus user could easily find the full matched entry in this page. After clicking the “taxol” in “Name & synonyms” column, molecule information page of taxol will be visible, where molecule structure, pharmacological and molecular properties, anticancer activities, targets, related herbs, references, etc., were well organized and displayed. In addition, user can easily visit other pages such as the information of herbs and targets by the inner hyperlink provided in this page (Fig. 3). The search for other keywords option can be similarly done as mentioned above. In addition, a case study was provided in CancerHSP to illustrate how to use this database to help uncover the underlying anticancer mechanisms of natural anticancer compounds (http://lsp.nwsuaf.edu.cn/load_intro.php?site=CancerHSP&id=60).

#### Browse

We have also introduced a powerful browsing option, which can provide an overall view on cell line data. Similar to result page of searching, the browsing page also allows users to sort and filter the result in every column of table. The primary organ or tissue sites of cell lines were provided in this page. In addition, the cell lines in browsing page are internal linked to result page of searching and partially external linked to Cancer Cell Line Encyclopedia (CCLE) database (Fig. 3), where the users could obtain more detailed information of the cell lines.

#### Download and update

As a public-facing database, CancerHSP provides a “Download” page where the entire database can be downloaded for further analysis. To maintain CancerHSP comprehensive and up to date, a submission interface was provided where users can submit his/her own data concerning to natural products for cancer treatment. However, to ensure accuracy of the submission, we will scrutinize the authentication of them.

## Discussion

To date, increasing herbal anticancer products were identified, isolated and characterized from herbs in last decades and constitute a substantial percentage of today’s new drugs. Unfortunately, most of these drugs were impeded by the major problems including narrow therapeutic index, strong side effects and drug resistance, which could reduce life quality of cancer survivors^13^. The most important reasons are unknown targets, poor absorption or rapid metabolism or excretion of a drug, resulting in unintended off-targets and low drug concentration levels in serum^14^,^15^. One of the useful way to develop a successful anticancer drug is to gather these information when the lead compound was found. However, to our knowledge, there is no such repository have been developed so far, which systematically provides information of anticancer herbs, anticancer ingredients, targets and ADME parameters of each ingredient. Inspired by the process of absorbed drugs and success of systems pharmacology (Fig. 1), we have developed CancerHSP which is the first step on this direction for systematic collection and evaluation of efficient natural products for cancer.

CancerHSP integrates large scale experimental studies and computational analyses to provide a mechanistic understanding of drug action across multiple scales of complexity ranging from molecular and cellular levels to tissue and organism levels. It can be used to identify targets for new drugs, study drug repositioning, and evaluate drug side effects and adverse events. Availability of natural products in the database for development of innovative combination cancer therapy regimens will provide a benchmark for the resolution of the cancer therapy translational research enterprise.

In all cancer treatment cases, the effectiveness of the treatment is directly related to the therapeutic ability to targets and to kill the cancer cells while affecting as few healthy cells as possible^16^. The data concerning to effect on healthy cells are also of importance. But there are insufficient data concerning to drug effects on normal cells and whole body, such as normal cells inhibit rate and life prolong rate in the current release of CancerHSP, due to the limitation of current research articles. We hope that much more work could be done for these kinds of data when an anticancer product was identified. Although we have included the most recent data from literature in CancerHSP, in order to maintain it comprehensive and up to date, we will incorporate the new data as soon as they will be available, and plan to update CancerHSP annually to incorporate new functions. In the future versions, more anticancer compounds and experimental information will be included, such as the drug sensitive, drug-drug interactions, drug metabolites and drug toxicities.

## Methods

### Data collection

In order to develop a comprehensive information resource of anticancer herbs and ingredients, research articles providing information related to keywords such as “herb”, “traditional Chinese medicine”, etc., coupled with keywords like “anticancer”, “cytotoxicity”, “anti-proliferation” and “apoptosis” were extracted from PubMed and Google scholar (as of 30^th^ 10, 2014). After careful reading these articles, information related to herbs, herbal ingredients and anticancer activities based on cancer cell lines were manually extracted and compiled. Further, the primary sites of cell lines and external links to CCLE^17^ were integrated into CancerHSP.

### Molecular information

The molecular structures of herbal ingredients were downloaded from PubChem^18^ and ChemSpider^19^, or produced by ISIS Draw 2.5 (MDL Information Systems, Inc.) and further optimized by Sybyl 6.9 (Tripos, Inc.) with Sybyl force field and default parameters^20^. InChIKey, a fixed length (25 character) condensed digital representation of the InChI (IUPAC International Chemical Identifier), was produced by Open Babel^21^ for each molecule. Further, for keeping uniformity, different format types of the chemical files were converted to SDF format by Open Babel, and the duplicates were removed according to InChIKey^22^. To facilitate searching, synonyms and CAS numbers for each molecule were obtained from PubChem and ChemSpider and included to CancerHSP.

### Drug targets

Drug targeting was performed by two in-house tools: SysDT and WES. SysDT is a systematic tool that efficiently integrates the chemical, genomic, and pharmacological information for drug targeting and discovery on a large scale, based on two powerful methods of random forest and support vector machine^8^. WES is a novel computational model which has been constructed to detect drug direct targets on a large scale based on the newly developed weighted ensemble similarity method. In order to strictly assess the relationships between compounds and corresponding targets, the mode of action was predicted by another in-house tool preAM, which is an accurate model to classify drug-target interactions into different action modes, i.e. activation and inhibition, based on the robust random forest algorithm.

### ADME properties

Analysis of ADME related pharmacokinetic properties, such as oral bioavailability (OB) and Caco-2 permeability (Caco-2), blood-brain barrier (BBB) and Lipinski’s rule of five (MW, AlogP, TPSA , Hdon, Hacc, RBN) were performed as previously described^22^. Detailed parameters’ information and calculation can be obtained from CancerHSP.

### Database framework and web interface

**Figure 4:**
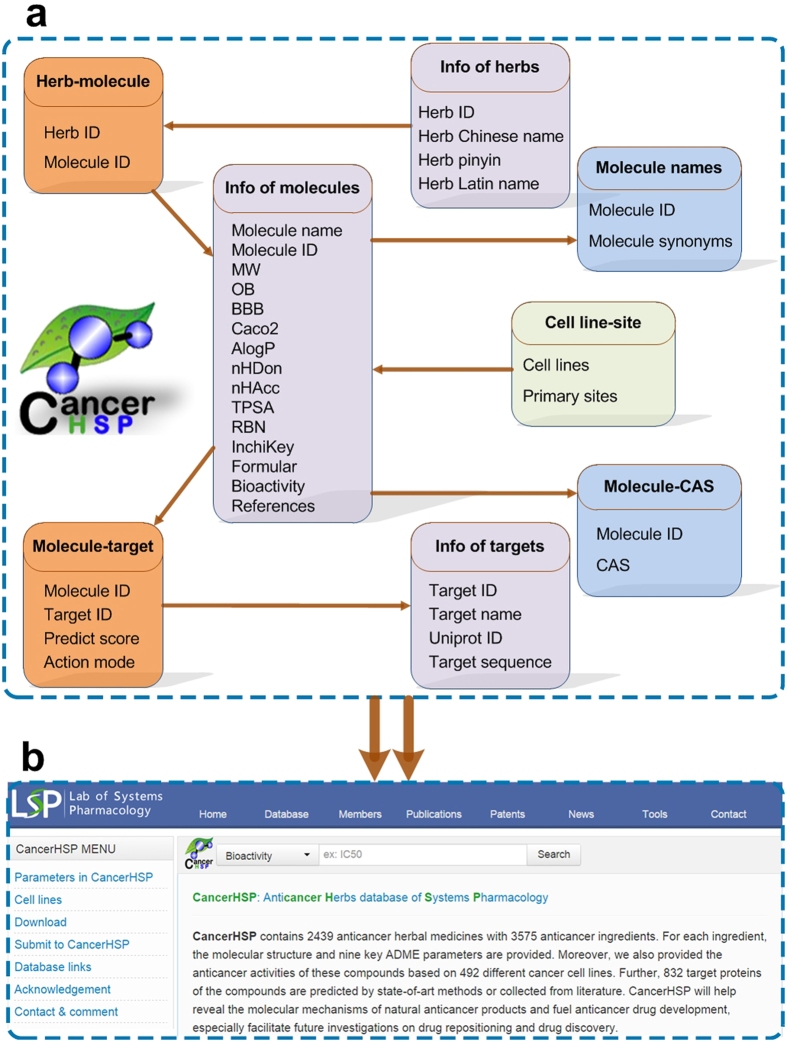
Overview of CancerHSP. (**a**) The back end is a MySQL database which consists of 8 parts, i.e., “Info of herbs” (herb ID and herb names), “Herb-molecule” (anticancer ingredients in each herb), “Info of molecules” (pharmacokinetic and pharmacological properties, bioactivity and references), “Molecule names” (synonyms of ingredients’ name), “Molecule-CAS” (CAS registry number of chemicals), “Molecule-target” (molecule-target relationships with action modes), “Info of targets” (target information) and “Cell line-site” (the primary sites of cell lines). (**b**) The front end is a web interface based on HTML, CSS, PHP and JavaScript programming languages.

CancerHSP is designed as a relational database on an apache server. All data were organized in a publicly available MySQL database as the back end (Fig. 4a), with a user-friendly web interface based on HTML, CSS, PHP and JavaScript programming languages as the front end (Fig. 4b).

## Additional Information

**How to cite this article**: Tao, W. *et al*. CancerHSP: anticancer herbs database of systems pharmacology. *Sci. Rep*. **5**, 11481; doi: 10.1038/srep11481 (2015).
